# Working while Caring for Older Adults with Hearing Impairment and Dementia: Evidence from the NSOC

**DOI:** 10.1093/geroni/igab046.1689

**Published:** 2021-12-17

**Authors:** Varshini Varadaraj, Bonnielin Swenor, Nicholas Reed, Emmanuel Garcia Morales

**Affiliations:** 1 The Wilmer Eye Institute, Baltimore, Maryland, United States; 2 Johns Hopkins School of Medicine, Baltimore, Maryland, United States; 3 Johns Hopkins Bloomberg School of Public Health, Baltimore, Maryland, United States

## Abstract

Age-related hearing loss (HL) and dementia are common among older adults. The implications of caregiving for older adults with dementia is documented. Whether the presence of HL modifies these association is unknown. We used data from the 2011 NHATS/NSOC. Hearing loss and dementia were identified among care recipients (CR). Our outcomes included: hours of care provided, and caregiver’s work activities. Among 1,013 caregivers, 456 assisted individuals without HL or dementia (HL-/D-), 229 with dementia (D+), 193 with HL, and 135 with HL and dementia (HL+/D+). In fully adjusted models, as compared to caregivers of HL-/D-, caregivers of D+ spent 39.1 hours more (95% CI: 13.6,64.6) in caregiving, caregivers of HL+/D+ spent 56.6 more hours (95% CI: 25.1,88.1). We found no differences in work activities between CR groups. The presence of HL increases the caregiving needs of adults with dementia. The additional time does not affect the labor participation of caregivers.

